# Meta-Analysis on the Association of C-Reactive Protein Polymorphisms with Metabolic Syndrome

**DOI:** 10.1055/s-0040-1710548

**Published:** 2020-07-09

**Authors:** Seyedeh Maryam Sharafi, Manijeh Mahdavi, Roya Riahi, Majid Kheirollahi, Roya Kelishadi

**Affiliations:** 1 Environment Research Center, Research Institute for Primordial Prevention of Non-Communicable Disease, Isfahan University of Medical Sciences, Isfahan, Iran; 2 Pediatric Inherited Diseases Research Center, Research Institute for Primordial Prevention of Non-Communicable Disease, Isfahan University of Medical Sciences, Isfahan, Iran; 3 Child Growth and Development Research Center, Research Institute for Primordial Prevention of Non-Communicable Disease, Isfahan University of Medical Sciences, Isfahan, Iran; 4 Department of Genetics and Molecular Biology, School of Medicine, Isfahan University of Medical Sciences, Isfahan, Iran

**Keywords:** C-reactive protein, single-nucleotide polymorphisms (SNPs), metabolic syndrome, meta-analysis

## Abstract

Polymorphisms in the C-reactive protein (CRP) genes might have crucial role in the development of metabolic syndrome (MetS). In the current comprehensive meta-analyses, we aim to provide a quantitative assessment of the association between CRP single-nucleotide polymorphisms (SNPs) and the risk of MetS. An electronic search was performed on several databases. After data extraction, random effect model was used to calculate the pooled odds ratio (OR) and 95% confidence intervals (CIs). Four independent studies including case–control, cohort, and cross-sectional methods were analyzed. Our meta-analysis indicated that CRP polymorphisms are not significantly associated with MetS (OR = 0.92, 95% CI = 0.77–1.10) with significant heterogeneity ( *I* ^2^ = 55.4%; *p* -value = 0.008). The subgroup analysis revealed that only GG has significant association with MetS (OR = 0.32, 95% CI = 0.13–0.80, *p* -value = 0.015) without significant heterogeneity ( *I* ^2^ = 0%, *p* -value > 0.05). In conclusion, this meta-analysis provides strong evidence that only some SNPs of CRP gene are associated with the risk for development of MetS; and this relationship does not exist in different ethnic populations.

## Introduction

Metabolic syndrome (MetS) is a growing public health problem worldwide. It is defined as a complex syndrome with coexistence of multiple cardiovascular risk factors consisting of atherogenic dyslipidemia, central obesity, proinflammatory and prothrombotic states, hypertension, and hyperglycemia. 1 2 It is associated with endothelial dysfunction, angiogenesis that might lead to increased risk for diabetes mellitus, insulin resistance, and cardiovascular diseases. 3

Ridker et al reported slight increase in moderate C-reactive protein (CRP) levels in relation to the number of MetS components in a follow-up study on healthy American women. 4 CRP consists of five 23 kDa subunits which has a crucial role in the human immune system. 5 Also, CRP largely regulated by interleukin 6, has been broadly used to evaluate various inflammatory states. 6 7 High concentrations of CRP are related with increased risk of the MetS. 2 Given that CRP is found to have an important role in MetS, the association between polymorphisms of inflammatory marker gene and MetS were studied. 3 Among various gene association studies, one of the most reported genes is CRP. The human CRP gene is on chromosome 1q21–1q23, approximately 1.9 KB, and has two exons, which are linked by a 280 base pair intron. 8

Many recent studies evaluated the genetic association between CRP single-nucleotide polymorphisms (SNPs) and MetS in different populations. 2 9 10 11 12 13 14 15 16 Some studies showed the association of CRP polymorphisms, 10 12 14 whereas some other studies did not. 11 13 Therefore, a comprehensive systematic review and meta-analysis are essential to evaluate the relationships between CRP polymorphisms and MetS. The present study aimed to define the association between MetS and the polymorphisms of the CRP gene: rs1800947, rs3093068, rs1205, and rs3091244.

## Methods

### Search Strategy

The electronic databases, such as Science Direct, PubMed, Web of Knowledge, Scopus, and Springer, were systematically searched for all available articles, without any limitation for publication date, language, article type, access type, and species until January of 2019. Articles with the following search terms in the titles, abstracts, or keywords were included: (((“C-reactive protein” OR CRP)) AND (metabolic syndrome OR MetS)) AND polymorphism. We also improved the search by revising the reference lists of all of the selected publications and finding supplementary documents related to these articles.

### Inclusion and Exclusion Criteria

For identifying suitable articles, the following criteria were included in this meta-analysis study. Any article published as an original study that considered the association between CRP polymorphisms and MetS. The available data for each polymorphism was reported and also necessary data to estimate the odds ratio (OR) and 95% confidence interval (CI) was provided. Furthermore, the reviews, animal studies, comments or editorials, overlapped articles, or studies with insufficient information on genotypes or allele frequencies were excluded.

### Data Extraction

The original information of search articles were extracted by two authors (M.M. and M.S.) independently using a reliable and standardized method. The following information was collected from each study: first author, year of publication, origin of the subjects, study design, variables adjusted for the analysis, and the Hardy–Weinberg equilibrium value of the overall association for each SNP of CRP gene.

### Statistical Analysis

The association between polymorphisms of CRP gene and MetS was evaluated. Statistical analysis was performed by pooled ORs and associated 95% CIs. The *Z* test was used for determining the significance of the pooled OR. Statistical analyses were performed by STATA software (version 12.0; Stata Corp LP, College Station, Texas, United States). A *p* -value of < 0.05 was considered as statistically significant. The heterogeneity among studies was detected using the chi-square test-based *Q* statistic, and then it was quantified using the *I* ^2^ statistic.

The Mantel–Haenszel fixed-effects model was considered when heterogeneity was not significant (i.e., *p* > 0.05 and *I* ^2^ < 50%). When heterogeneity was significant, random-effects model (the DerSimonian–Laird method) was used. Finally, for evaluating the existence of heterogeneity among groups, sensitivity analyses were conducted. In addition to calculating publication bias, the Begg's and Egger's tests were applied, in which *p* -values of less than 0.05 indicate significant publication bias.

## Results

### Characteristics of the Included Studies

**Fig. 1:**
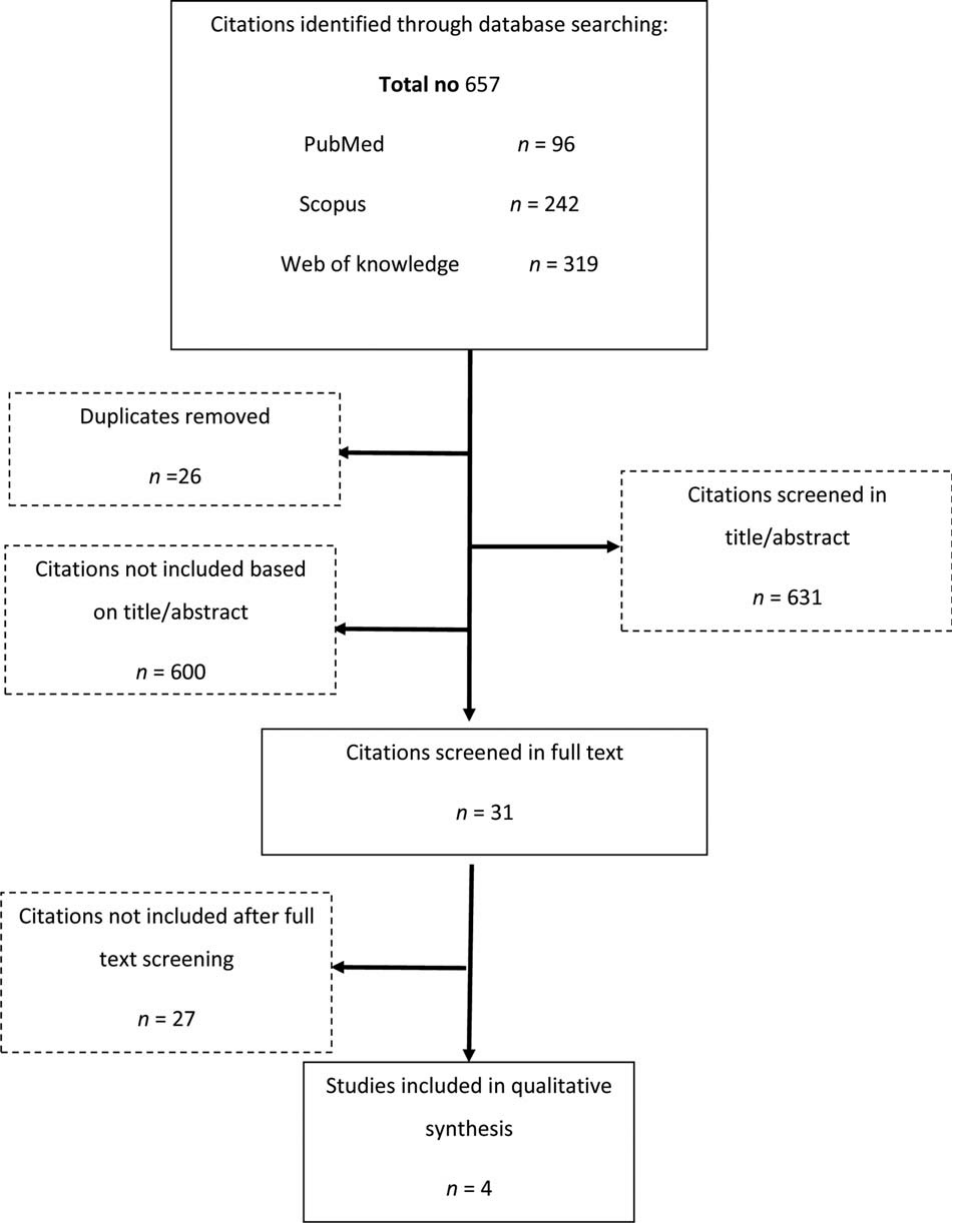
The methods for selection process.

Detailed flowchart for this study searching is presented in Fig. 1 . According to the aforementioned search term, 657 potentially appropriate articles were identified. After removing 26 duplicates, 631 articles remained for the next step. Among them, 31 papers were selected for reviewing the full text. In total, 27 publications were omitted mainly because they had no relation or meeting abstracts or reviews. In conclusion, four full-text articles were included in the present meta-analysis study. The main characteristics of the studies included in this meta-analysis are listed in Table 1 . As shown in the table, there are four articles containing two case–control studies from Egypt 10 and Iran, 13 one cross-sectional study from Taiwan, 12 and one cohort study from the United Kingdom. 11

**Table 1:** Characteristics of included studies

**ID**	**Authors**	**Year**	**Country**	**SNPs**		**OR (95% CI)**	**Study design**	**Statistical analysis (HWE,** ***p*** -Value **)**
1	Tarek et al 10	2013	Egypt	1059 (rs1800947)	GC + CC	3.59 (1.30–9.93)	Case–control	*p* = 0.001
					C allele	3.37 (1.29–8.82)		*p* = 0.01
2	Hsu et al 12	2010	Taiwan	rs3091244		1.62 (1.05–2.49)	Cross-sectional	*p* = 0.029
3	Nikpour et al 13	2015	Iran	T allele	rs3091244	1.70 (0.98–2.96)	Case–control	*p* = 0.059
**ID**	**Study**	**Year**	**Country**	**SNPs**		**Population (** ***N*** **event/case) / (** ***N*** **event/control)**	**Study design**	**Statistical analysis (HWE,** ***p*** -Value **)**
4	Darya et al 11	2011	UK	rs1205		(618/2100)/(141/1875)	Cohort	*p* = 0.59
				rs3093068		(616/2093)/(141/1875)		*p* = 0.88

Abbreviations: CI, confidence interval; HWE, Hardy–Weinberg equilibrium; OR, odds ratio; SNP, single-nucleotide polymorphism.

### Meta-Analysis Results

**Fig. 2:**
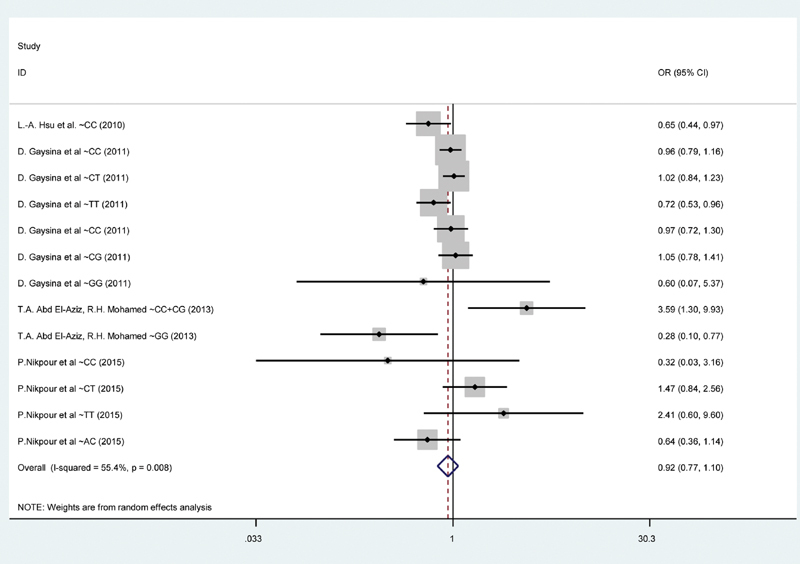
The forest plot of the association between C-reactive protein (CRP) gene polymorphism with metabolic syndrome ( *n* = 4, all studies).

**Fig. 3:**
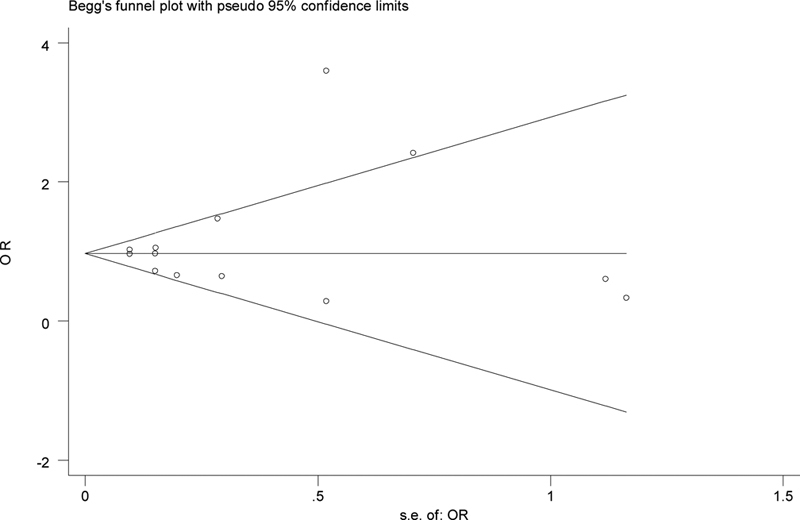
The funnel plot of the association between C-reactive protein (CRP) gene polymorphism with childhood and adolescence metabolic syndrome ( *n* = 4, all studies). OR, odds ratio.

The forest plot for association between CRP gene polymorphism with MetS according to all the studies is shown in Fig. 2 . Overall, there was no significant association between CRP gene polymorphism and MetS (OR = 0.92, 95% CI = 0.77–1.10); as shown in Fig. 3 , significant heterogeneity was documented ( *I* ^2^ = 55.4%; *p* -value = 0.008). We used funnel plot and Egger's test to assess the publication bias. Based on both funnel plot ( Fig. 3 ) and Egger's test there was no evidence of publication bias (for all studies: *p* = 0.44).

**Fig. 4:**
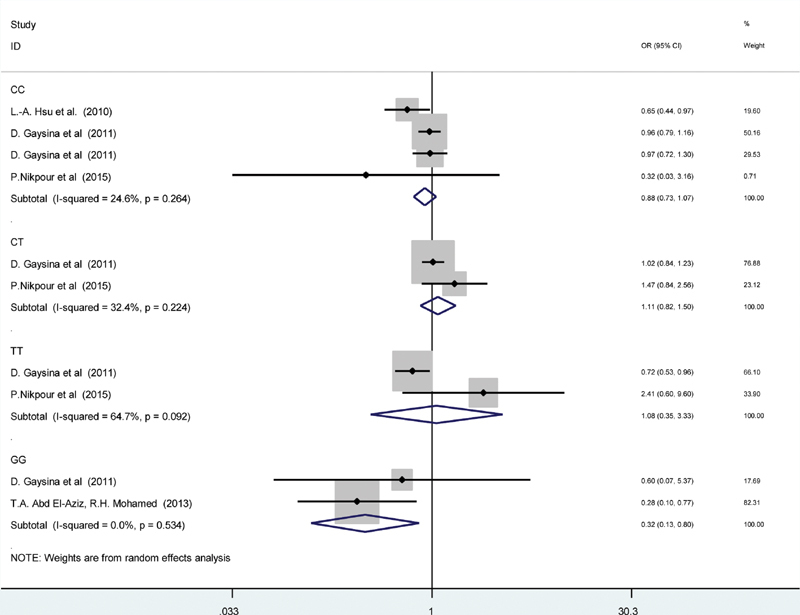
The forest plot of the association between C-reactive protein (CRP) gene polymorphism with childhood and adolescence metabolic syndrome according to the genotype subgroups.

Result of subgroup meta-analysis according to genotype is shown in Fig. 4 . In the GG subgroup, the association of CRP gene polymorphism with MetS was significant (OR = 0.32, 95% CI = 0.13–0.80, *p* -value = 0.01) with no significant heterogeneity ( *I* ^2^ = 0%, *p* -value = 0.534) ( Fig. 4 ). It is followed by performing sensitivity analysis in which the individual studies were removed sequentially to assess their effect on the results. There was no difference from the initial analysis. Therefore, it is suggested that the results of this meta-analysis were strong.

## Discussion

Various investigations have reported the association of CRP polymorphisms and MetS; however, the findings of previous studies have been controversial. To start, we chose the CRP gene because there is strong evidence that it is a powerful predictor of incident MetS events. As a result, the most common variants studied were the following polymorphisms: rs1800947, rs3093068, rs3091244, and rs1205 of the CRP gene. In the current research work, to prove whether CRP polymorphism is responsible for the development of MetS, we conducted a meta-analysis, which included four independent studies. Based on literature search, this article is the first comprehensive meta-analysis to evaluate the possible correlations of CRP polymorphisms in MetS.

In adult populations, the association between the CRP rs3091244 and MetS has been evaluated in several studies. 9 12 In the present study, we assessed the association of different variants of the CRP gene with MetS. The main results indicated that the CRP polymorphisms including rs1800947, rs3093068, rs3091244, and rs1205 were not related with MetS. The lack of association could be described by the limited number of studies. Consequently, it is essential to increase the number of studies to have a substantial outcome. Additionally, in subgroup analysis, it was indicated that the GG subgroup of the mentioned CRP gene polymorphism has significant association with metabolic syndrome.

Some studies reported the association between different populations. For example, in 2010, Hsu et al demonstrated the correlation of rs3091244 on the risk of MetS in Taiwanese adults. This study showed that the non-CC genotypes are associated significantly with MetS after adjustment for smoking, age, sex, and body mass index. 12 Similarly, in our study, among the non-CC genotypes only GG had significant association. But other studies failed to confirm this association. 11 13 Nikpour et al reported that in Iranian children and adolescents, −286C > A > T CRP polymorphism (rs3091244) is not associated with the increased risk for MetS. 13 Furthermore, Gaysina et al did not find association between CRP gene variants rs1205 and rs3093068 with the metabolic syndrome in U.K. population. 11

Some limitations of our meta-analysis study should be noted. First, the limited number of articles for most of the CRP polymorphisms was available. Second, the languages of the publications were limited to English. Third, our meta-analysis was based on evaluations without adjusting the data for population characteristics such as ethnicity, age, etc. Fourth, due to lack of access to detailed data used in studies, our meta-analyses are based on the various data of CRP SNPs, and finally, it must be noted that the study design of included articles in this meta-analysis was different ( Table 1 ).

Despite these limitations, the current study also has some advantages. First, the statistical power of the analyses was considerably increased as a large number of samples were derived from different studies. Second, in our analyses, we investigated different ethnicity population.

## Conclusion

In summary, this meta-analysis demonstrated considerable evidence that there were no correlations between various polymorphisms of CRP gene (rs1800947, rs3093068, rs3091244, and rs1205) and the risk of MetS in various populations. Moreover, it is recommended that future researches into the mutual effects of the environment and genes may improve existing concept of the associations between polymorphisms of CRP gene and developing MetS. Further work will help explain the medical and biotechnological implications of these associations.
